# Decreased VEGF-A and sustained PEDF expression in a human retinal pigment epithelium cell line cultured under hypothermia

**DOI:** 10.1186/s40659-015-0034-7

**Published:** 2015-07-30

**Authors:** Masayuki Takeyama, Masahiko Yoneda, Masahiko Gosho, Masayoshi Iwaki, Masahiro Zako

**Affiliations:** Department of Ophthalmology, Aichi Medical University, Nagakute, 480-1195 Aichi Japan; Department of Biochemistry and Molecular Biology, School of Nursing and Health, Aichi Prefectural University, Nagoya, 463-8502 Aichi Japan; Department of Clinical Trial and Clinical Epidemiology, Faculty of Medicine, University of Tsukuba, Tsukuba, 305-8575 Ibaraki Japan

**Keywords:** Age-related macular degeneration (AMD), Hypothermia, Pigment epithelium–derived factor (PEDF), Retinal pigment epithelium (RPE), Vascular endothelial growth factor (VEGF), Vitrectomy

## Abstract

**Background:**

Previous reports have described a decrease in retinal temperature and clinical improvement of wet age-related macular degeneration (AMD) after vitrectomy. We hypothesized that the retinal temperature decrease after vitrectomy plays a part in the suppression of wet AMD development. To test this hypothesis, we evaluated the temperature dependence of the expression of vascular endothelial growth factor-A (VEGF-A) and in vitro angiogenesis in retinal pigment epithelium (RPE).

**Results:**

We cultured ARPE-19 cells at 37, 35, 33 and 31°C and measured the expression of VEGF-A, VEGF-A splicing variants, and pigment epithelium–derived factor (PEDF). We performed an in vitro tube formation assay. The dehydrogenase activity was also evaluated at each temperature. Expression of VEGF-A significantly decreased with decreased temperature while PEDF expression did not. VEGF165 expression and in vitro angiogenesis also were temperature dependent. The dehydrogenase activity significantly decreased as the culture temperature decreased.

**Conclusions:**

RPE cultured under hypothermia that decreased cellular metabolism also had decreased VEGF-A and sustained PEDF expression, creating an anti-angiogenic environment. This mechanism may be associated with a beneficial effect after vitrectomy in patients with wet AMD.

## Background

Previous reports have shown that wet age-related macular degeneration (AMD) improves clinically after vitrectomy, even though the primary pathogenesis in wet AMD occurs in the outer retina, including the retinal pigment epithelium (RPE), Bruch’s membrane, and choroid [1–5]. Secretion by the RPE of vascular endothelial growth factor-A (VEGF-A), which acts as an angiogenic molecule in the retina, plays a critical role in the development of wet AMD [6–9]. Vitreous oxygenation, vitreoretinal traction removal, and diffusion of vitreous substances are assumed to contribute to the beneficial effects [10–12]; however, the precise mechanism by which vitrectomy affects wet AMD is still unclear.

The intraocular temperature is influenced by the temperature of the outside air and modified by intraocular surgery. The temperature within the anterior chamber is most affected by the outside air. Romano et al. measured and compared intraocular temperatures during surgery and found that the temperature within the anterior chamber was 23.6°C [13]. The retinal temperatures immediately adjacent to the fovea before and after vitrectomy were 34.9 and 32.6°C, respectively [14]. Retinal temperature just after vitrectomy is 2.3°C lower than before vitrectomy with the eyelid open, suggesting that the difference may be induced by cooling mediated by outside air and an anatomical alteration in the vitreous cavity after vitrectomy.

This decrease in retinal temperature after vitrectomy may have a beneficial effect on wet AMD. It was previously shown that secretion of VEGF-A by RPE at 34°C was decreased compared with secretion by cells grown at 37°C [15]; however, that study compared VEGF-A secretion only at the two temperatures, which might not give a complete overview of changes in VEGF-A expression at various temperatures that are clinically relevant after vitrectomy. Here we cultured RPE at 37, 35, 33, and 31°C and measured the expression of VEGF-A, VEGF-A splicing variants, and placental growth factor (PlGF). Furthermore, we measured the expression of pigment epithelium–derived factor (PEDF), which is a major angiogenesis inhibitor in retina [6, 16–18]. To investigate differences that may occur in the angiogenic environment upon temperature variation, we also performed an in vitro tube formation assay using the conditioned medium collected after culture at each temperature. Dehydrogenase activity was measured to evaluate cellular metabolism at each temperature. Finally, our results support the hypothesis that the continuous mild hypothermia of retina achieved after vitrectomy may have a beneficial effect in wet AMD.

## Results and discussion

### VEGF-A

**Fig. 1:**
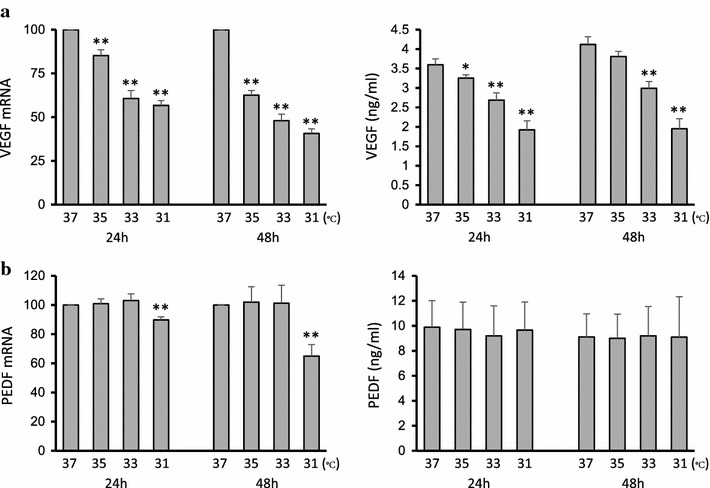
Temperature-dependent decrease in VEGF-A expression and sustained, temperature-independent PEDF expression in ARPE-19 cells. Expression of mRNA and protein abundance were measured by real-time PCR and ELISA, respectively. **a** VEGF-A expression showed a statistically significant temperature-dependent decrease. **b** PEDF expression showed no significant change within the temperature range from 37 to 31°C, with the exception of mRNA at 31°C. Data are shown as mean ± SD (*n* = 4). *P < 0.05, **P < 0.01.

VEGF-A mRNA expression was measured by real-time PCR. In samples collected after 24 h, the mRNA expression measured at lower temperatures was decreased compared to expression in cells cultured at 37°C, with an observed decrease of 14.8% at 35°C, 39.3% at 33°C, and 43.3% at 31°C. In samples collected after 48 h, compared with cells cultured at 37°C, the decreases measured at each reduced temperature were 35°C, 37.3%; 33°C, 52.0%; and 31°C, 59.3%. This temperature-dependent decrease was statistically significant (Fig. 1a, left).

VEGF-A protein concentration in the conditioned medium was measured by enzyme-linked immunosorbent assay (ELISA). In samples collected after 24 h, the decreases measured at each reduced temperature compared to 37°C were as follows: 35°C, 9.55%; 33°C, 25.3%; and 31°C, 46.6%. In samples collected after 48 h, the decreases measured at each reduced temperature were 35°C, 7.52%; 33°C, 27.3%; and 31°C, 52.6%. Significant temperature-dependent decreases in protein abundance were also observed (Fig. 1a, right). Overall, expression of both VEGF-A mRNA and protein decreased as the culture temperature decreased in samples collected after either 24 or 48 h.

### PEDF

PEDF mRNA expression was measured by real-time PCR. In samples collected after 24 h, no significant change in PEDF mRNA expression was found over the range from 37–33°C. However, PEDF mRNA expression at 31°C was significantly lower than at 37°C. Similarly, in samples collected after 48 h, no significant expression change was found over the range from 37–33°C, but at 31°C, expression significantly decreased by 45.0% compared with expression in cells cultured at 37°C (Fig. 1b, left). The concentration of PEDF protein in the conditioned medium was measured by ELISA. PEDF protein abundance showed no significant change over the range of 37–31°C (Fig. 1b, right).

### VEGF165

**Fig. 2:**
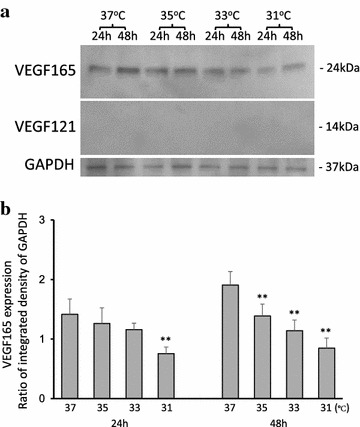
Western blot analysis of VEGF165 and VEGF121. **a** Temperature-dependent decrease in VEGF165 expression shown in samples collected after 24 and 48 h of incubation at the specified temperature. VEGF121 was not detected in any of the samples examined. **b** Quantitative analysis of VEGF165 abundance. Data are mean ± SD (*n* = 4); **P < 0.01.

VEGF165 expression in conditioned medium was measured by western blot analysis, and the results are shown in Fig. 2. In samples collected after 24 h, decreased expression was observed at all temperatures compared to expression at 37°C, with a percent decrease of 10.7% at 35°C; 12.7% at 33°C; and 39.9% at 31°C. In samples collected after 48 h, the corresponding decreases were 35°C, 6.41%; 33°C, 15.7%; and 31°C, 42.3%. Expression at 31°C was significantly decreased in samples collected after 24 and 48 h. Generally, expression was observed to decrease in a temperature-dependent fashion.

### VEGF121

VEGF121 expression was not detected by western blot analysis in any of the samples examined (Fig. 2a).

### PlGF

PlGF expression was not detected by real-time PCR or ELISA in any of the samples examined (*data not shown*).

### Tube formation assay

**Fig. 3:**
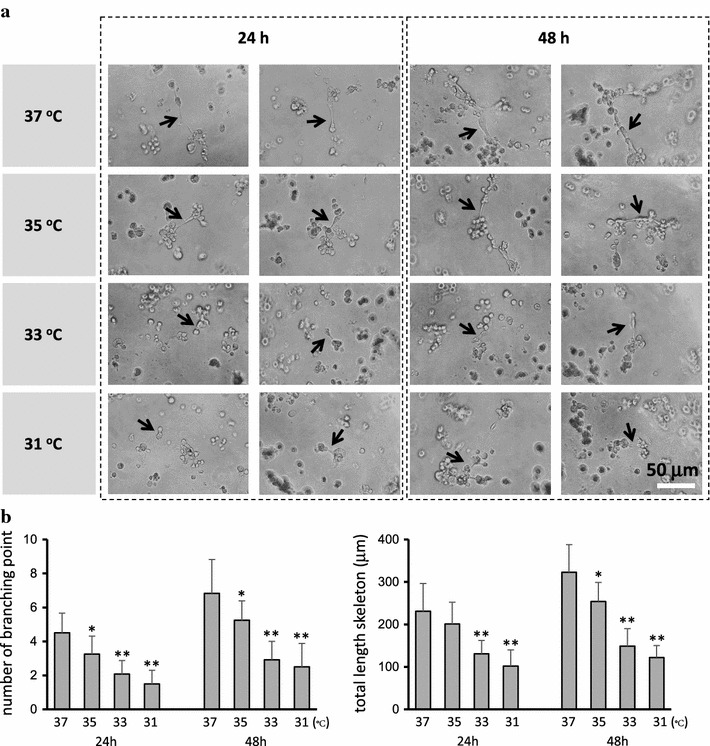
Angiogenesis assay measuring tube formation ability of HUVECs with conditioned medium from ARPE-19 cells. **a** Representative photomicrographs. Typical tube formation was not seen in any of the samples with conditioned medium collected from cells cultured at temperatures from 37 to 31°C after 24 and 48 h. Significant differences were observed in the number of branching points and total skeleton length (*arrows*) among each of the conditions. *Bar* 50 μm. **b** Quantitative analysis of the number of branching points and total skeleton length. Data are presented as mean ± SD (*n* = 4). *P < 0.05, **P < 0.01.

To investigate possible changes in the angiogenic environment produced by RPE, we performed endothelial tube formation (in vitro angiogenesis) assays using conditioned medium from ARPE-19 cells cultured at various temperatures, from 37 to 31°C, and for either 24 or 48 h. Representative photomicrographs and image quantitative analysis are shown in Fig. 3. Typical tube formation was not found in any of the samples. However, significant differences in the number of branching points and total skeleton length were observed among the conditions. With conditioned medium samples collected after 24 h, the number of branching points was reduced compared to the values observed at 37°C by the following percentages: at 35°C, 26.6%; at 33°C, 56.5%; and at 31°C, 60.9%. In samples collected after 48 h, the percent decrease in expression was as follows: at 35°C, 47.1%; at 33°C, 61.7%; and at 31°C, 73.8%. With respect to total skeleton length, in samples collected after 24 h and compared with conditioned medium from cells cultured at 37°C as the baseline, the decreases observed at each temperature were as follows: at 35°C, 19.3%; at 33°C, 44.6%; and at 31°C, 80.7%. In samples collected after 48 h, the decreases observed at each temperature were as follows: at 35°C, 14.4%; at 33°C, 47.1%; and at 31°C, 83.6%. Overall, the number of branching points and total skeleton length in samples collected after either 24 h or 48 h showed significant decreases as the culture temperature decreased.

### Measurement of cellular metabolism

**Fig. 4:**
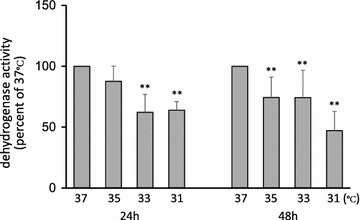
Dehydrogenase activity in ARPE-19 cells at each adjusted temperature. Cellular metabolism evaluated by dehydrogenase activity significantly decreased as the culture temperature decreased. Data are presented as mean ± SD (*n* = 5); **P < 0.01.

The metabolism of cells cultured at each adjusted temperature was evaluated by dehydrogenase activity. In samples collected after incubation for 24 h, the decreases measured at each reduced temperature compared to 37°C were as follows: 35°C, 12.3%; 33°C, 37.8%; and 31°C, 36.0%. In samples collected after incubation for 48 h, the decreases measured at each reduced temperature were 35°C, 25.6%; 33°C, 25.7%; and 31°C, 52.8%. Significant temperature-dependent decreases in dehydrogenase activity were observed in samples collected after either 24 or 48 h (Fig. 4).

The balance between VEGF-A and PEDF plays an important role in choroidal neovascularization [6, 16–18]. In the present study, VEGF-A expression significantly decreased in RPE with temperature dependence, as analyzed using real-time PCR and ELISA. Western blot analysis showed a temperature-dependent decrease in VEGF165 expression. In contrast, PEDF protein abundance did not show significant change. Thus, RPE cultured at temperatures lower than 37°C may exhibit an anti-angiogenic environment because of the decreased VEGF-A expression in conjunction with sustained PEDF expression. The results of the tube formation angiogenesis assay also supported this hypothesis, although typical tube formation was not observed, probably because of insufficient VEGF expression.

The sustained PEDF that is secreted from RPE predominantly to the apical side [19–22] may play a role in preventing wet AMD development by the following two mechanisms. First, because the secreted PEDF plays an autocrine role in maintaining RPE function [20], healthy RPE maintained by sufficient PEDF should prevent an invasion of choroidal neovascularization to the retinal side. Second, PEDF in the basal side, even if it is not a large amount, may act as an antagonist for VEGF-A. Becerra et al. investigated PEDF localization in monkey eye and detected PEDF not only in the apical side but also in the Bruch membrane [21].

The measurement of dehydrogenase activity at each adjusted temperature showed a cellular metabolism decrease in association with the culture temperature decrease. We confirmed no significant difference in the number of cells in the dish at the end of incubation between each sample (*data not shown*). This result implies that a decrease in VEGF-A expression is correlated with a decrease in metabolism in cells cultured under hypothermia, although it is still unclear why PEDF expression is not so closely correlated.

**Fig. 5:**
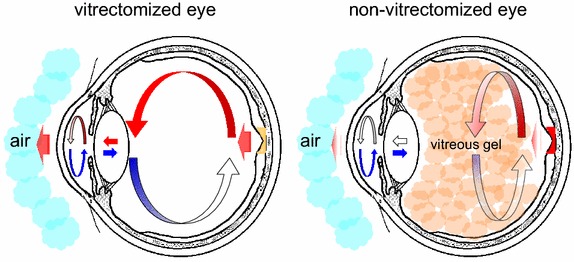
Hypothetical intraocular thermal diffusion in vitrectomized and non-vitrectomized eyes. *Red arrows* indicate the stream of heat within the eye globe. *Blue arrows* represent the stream of cooling by the outside air. Because of thermal diffusion from the posterior to anterior chamber through the lens, the anterior vitreous cavity temperature may be lower than the posterior vitreous cavity temperature. In the vitrectomized eye, without viscous vitreous, a more convective flow may occur because of the difference in temperature between the anterior and posterior vitreous cavities, compared to the situation with viscous vitreous in the non-vitrectomized eye, in which a convective flow may not easily occur.

The retinal temperature in a vitrectomized eye is lower than that in a non-vitrectomized eye, but this measurement of real-time retinal temperature in humans may be possible only during surgery. The anterior chamber temperature is remarkably cooled [13], and ophthalmologists often observe a warm current of aqueous humor convective flow caused by cooling of the anterior chamber by outside air. Because of thermal diffusion from the posterior to the anterior chamber through the lens, the anterior vitreous cavity temperature may be lower than the posterior vitreous cavity temperature. In the vitreous cavity not filled with viscous vitreous in a vitrectomized eye, a more convective flow may occur because of the difference in temperature between the anterior and posterior vitreous cavities; however, because of the viscous vitreous in a non-vitrectomized eye, such a convective flow may not easily occur. Our hypothesis regarding this intraocular thermal diffusion is shown in Fig. 5. The strict measurements taken of retinal temperature immediately adjacent to the fovea by Landers et al. during surgery were as follows: before vitrectomy, 34.9°C; at the end of vitrectomy after plugging the sclerotomies and closing the infusion line for 5 min, 32.6°C [14]. This difference of 2.3°C may have been enough to decrease VEGF-A expression in the eye, as our present results revealed that a 2°C decrease caused a significant decrease in VEGF-A expression in vitro.

## Conclusions

RPE cultured under hypothermia that decreased cellular metabolism exhibit decreased VEGF-A and sustained PEDF expression, creating an anti-angiogenic environment. This mechanism may be associated with a beneficial effect after vitrectomy in patients with wet AMD.

## Methods

### RPE culture in adjusted temperature

The human RPE cell line, ARPE-19, was obtained from the American Type Culture Collection (Manassas, VA, USA). Cultures were seeded with 5 × 10^5^ cells in 35-mm culture dishes with 2 ml of Dulbecco’s minimal essential medium (Invitrogen, Carlsbad, CA, USA) containing antibiotics (100 U/mL penicillin G and 100 mg/mL streptomycin sulfate; Invitrogen) and 10% fetal calf serum, and grown at 37°C in an atmosphere of 5% CO_2_. Subsequently, medium change was performed every 24 h. At day 2, cells reached confluence. At day 3, medium was changed with 2 ml of Dulbecco’s minimal essential medium containing antibiotics and 1% fetal calf serum. At day 4, medium was changed, and cells were transferred to temperature-adjusted incubators at 37, 35, 33 and 31°C in an atmosphere of 5% CO_2_. The ARPE-19 cell samples and conditioned media were collected at 24 h and 48 h after the start of incubation at each adjusted temperature. Conditioned media samples were stored at −80°C until use.

### Real-time PCR

Isolation of total RNA from ARPE-19 cells was performed using the SV Total RNA Isolation System (Promega, Madison, WI, USA) according to the manufacturer’s instructions. Isolated total RNA was reverse transcribed to DNA using random primers using a SuperScript^®^ VILO™ cDNA Synthesis Kit^®^ (Invitrogen), according to the manufacturer’s instructions. Real-time PCR was performed using the Thermal Cycler Dice^®^ Real Time System (Takara Bio Incorporated, Shiga, Japan), and SYBR^®^ Premix Ex Taq™ (Takara Bio Incorporated) was used for quantification of VEGF-A, PlGF, and PEDF mRNA. Thermal cycle conditions included an initial denaturation at 95°C for 10 s, followed by 40 cycles of PCR amplification (95°C for 15 s, 60°C for 1 min). The specificity of the amplification was confirmed using melting-curve analysis. Expression of the targeted mRNA was analyzed, with expression of glyceraldehyde-3-phosphate dehydrogenase (GAPDH) used for normalization. We analyzed these data with the competitive C_t_ (ΔΔC_t_) method according to the manufacturer’s instructions (Takara Bio Incorporated). Primer sequences are shown in Table 1.

**Table 1:** Primer pairs used for real-time PCR analysis

Gene (GenBank accession no.)	Direction	Oligonucleotide sequence (5′ → 3′)
VEGF-A (NM_001025366)	Sense	TCACAGGTACAGGGATGAGGACAC
	Antisense	TCCTGGGCAACTCAGAAGCA
PlGF (NM_002632.5)	Sense	GAGAGAAGCAGAGACCCACAGAC
	Antisense	GAGGCATTCAGCAGGGAAA
PEDF (NM_002615.5)	Sense	CCCATGATGTCGGACCCTAA
	Antisense	TGTCATGAATGAACTCGGAGGTG
GAPDH (NM_002046)	Sense	GCACCGTCAAGGCTGAGAAC
	Antisense	TGGTGAAGACGCCAGTGGA

### ELISA

Assays were performed using the Quantikine human VEGF ELISA Kit (R&D Systems, Minneapolis, MN), Quantikine human PlGF ELISA Kit (R&D Systems), and human PEDF ELISA (BioVendor Laboratory Medicine, Inc., Modrice, Czech Republic). An aliquot of 200 μl of conditioned medium was used per well.

### Western blot analysis

Conditioned medium (10 μl) was mixed with sodium dodecyl sulfate gel-loading buffer. Samples were loaded onto 15% sodium dodecyl sulfate polyacrylamide gels. After electrophoresis, proteins were electro-transferred to nitrocellulose membranes and developed with goat anti-human VEGF165 polyclonal antibody (1:1,000; R&D Systems) and polyclonal rabbit anti-goat immunoglobulins/horseradish peroxidase (HRP) (1:10,000; Dako, Denmark). The membranes were then incubated in stripping buffer (Thermo Fisher Scientific Inc., Waltham, MA), washed with phosphate-buffered saline containing 0.1% Tween-20 (Wako Pure Chemical Industries, Ltd., Osaka, Japan), and then developed a second time with rabbit anti-human VEGF121 polyclonal antibody (1:1,000; Rockland Immunochemicals, Inc., Gilbertsville, PA) and polyclonal swine anti-rabbit immunoglobulins/HRP (1:10,000; Dako). These data were normalized by GAPDH expression. The cells in each dish were lysed by 200 μl of CelLytic M (Sigma-Aldrich, St.Louis, MO, USA), and 10 μl of prepared cell lysate was used for the measurement of GAPDH expression. Polyclonal goat anti-GAPDH antibody (1:1,000; Santa Cruz Biotechnology, Inc., Santa Cruz, CA, USA) was used as primary antibody, and polyclonal rabbit anti-goat immunoglobulin/HRP (1:10,000; Dako) was used as secondary antibody. All western blots were finally developed by HRP with the detection reagent Western Lightning Plus ECL (PerkinElmer Inc., Waltham, MA). Band densities were quantified using ImageJ software (National Institute of Health, http://rsb.info.nih.gov/ij/).

### Tube formation assay

To examine the angiogenic environment produced by RPE, we performed in vitro tube formation assays using an in vitro angiogenesis assay kit (Merck Millipore, Billerica, MA, USA). The 96-well assay plate was prepared according to the manufacturer’s instructions. Human umbilical cord vein endothelial cells (HUVEC, Lonza, Basel, Switzerland, No. CC-2517) were plated (2.0 × 10^4^ cells/well) with 150 μl of the conditioned medium in a prepared 96-well plate, incubated at 37°C for 5 h, and photographed (field/well) under the microscope. On six photographs in each group, two independent observers manually counted the number of branching points and measured the length of the skeleton structure.

### Measurement of cellular metabolism

To investigate the metabolism of cells cultured at each adjusted temperature, we measured the dehydrogenase activity, which reflects cellular metabolism. ARPE-19 cells at 1 × 10^4^ cells/well were seeded in a 96-well plate with 100 μl of medium, and the schedule of medium changes was the same as described above. At the end of the culture period, the dehydrogenase activity was measured using a Cell Counting Kit-8 (Dojindo Laboratories, Kumamoto, Japan). According to the manufacturer’s instructions, we assumed the 450 nm absorbance as an index of the dehydrogenase activity.

### Statistical analysis

Experiments were repeated four times. The results are presented as the mean ± standard deviation (SD). Comparison of more than two groups was performed using analysis of variance, including temperature as a factor, with Dunnett’s test to adjust for multiplicity. The value measured at 37°C was defined as the control. We used SAS 9.3 software (SAS Institute, Cary, NC, USA) for all statistical analyses. Differences with P values <0.05 were considered significant.

## Abbreviations

AMD: age-related macular degeneration
ELISA: enzyme-linked immunosorbent assay
GAPDH: glyceraldehyde-3-phosphate dehydrogenase
HUVEC: human umbilical cord vein endothelial cell
PlGF: placental growth factor
PEDF: pigment epithelium–derived factor
RPE: retinal pigment epithelium
VEGF-A: vascular endothelial growth factor-A
